# Changes in crop trait plasticity with domestication history: Management practices matter

**DOI:** 10.1002/ece3.10690

**Published:** 2023-11-15

**Authors:** Victoria Nimmo, Cyrille Violle, Martin Entz, Andres G. Rolhauser, Marney E. Isaac

**Affiliations:** ^1^ University of Toronto Toronto Ontario Canada; ^2^ CEFE, Univ. Montpellier, CNRS, EPHE, IRD Montpellier France; ^3^ University of Manitoba Winnipeg Manitoba Canada; ^4^ University of Toronto Scarborough Toronto Ontario Canada; ^5^ Departamento de Métodos Cuantitativos y Sistemas de Información, Facultad de Agronomía Universidad de Buenos Aires Buenos Aires Argentina; ^6^ IFEVA, CONICET, Facultad de Agronomía Universidad de Buenos Aires Buenos Aires Argentina

**Keywords:** agroecology, functional traits, intercropping, organic agriculture, trait space, wheat

## Abstract

Crop domestication has led to the development of distinct trait syndromes, a series of constrained plant trait trade‐offs to maximize yield in high‐input agricultural environments, and potentially constrained trait plasticity. Yet, with the ongoing transition to organic and diversified agroecosystems, which create more heterogeneous nutrient availability, this constrained plasticity, especially in root functional traits, may be undesirable for nutrient acquisition. Such agricultural systems require a nuanced understanding of the soil‐crop continuum under organic amendments and with intercropping, and the role crop genetic resources play in governing nutrient management and design. In this study, we use a functional traits lens to determine if crops with a range of domestication histories express different functional trait plasticity and how this expression changes with soil amendments and intercropping. We utilize a common garden experiment including five wheat (*Triticum aestivum*) varietals with a range of domestication histories planted in a factorial combination with amendment type (organic and inorganic) and cropping design (monoculture or intercropped with soybean). We use bivariate, multivariate and trait space analyses to quantify trait variation and plasticity in five leaf and five root functional traits. Almost all leaf and root traits varied among varieties. Yet, amendment type was nearly inconsequential for explaining trait expression across varieties. However, intercropping was linked to significant differences in root acquisitive strategies, regardless of the varietals' distinct history. Our findings show substantial leaf and root trait plasticity, with roots expressing greater trait space occupation with domestication, but also the strong role of management in crop trait expression. We underscore the utility of a functional trait‐based approach to understand plant–soil dynamics with organic amendments, as well as the role of crop genetic histories in the successful transition to low‐input and diversified agroecosystems.

## Graphical abstract

We performed a common garden experiment with five wheat (*Triticum aestivum*) varietals spanning 150 years of domestication history. Wheat was planted with either inorganic or organic soil amendments and under monocropping or intercropping with soybean (*Glycine max*). Our findings show roots expressing greater trait space occupation with domestication but also the strong role of management in crop functional trait expression.

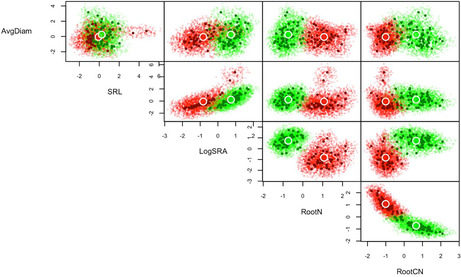

## 1 INTRODUCTION

Crop domestication has pursued a nearly singular focus on maximizing yield‐related traits, with a focus on yields specifically within high‐input agricultural environments since the Green Revolution. Such environments are centred on a surplus of available resources and a selection procedure for uniform aboveground phenotypic trait expression. Selection for crops that express certain reproductive and leaf traits has formed detectable domestication syndromes: suites of plant traits that differ between crops and their wild progenitors (Meyer et al., 2012). Through domestication, crops tend to express traits associated with higher rates of resource capture compared to their wild progenitors (Milla et al., 2014). Intraspecific variation can be formed from inherited differences and plasticity (Matesanz et al., 2012) and plays an important role in a plants' ability to adapt to changes in environmental conditions (Aspinwall et al., 2015). Broadly, trait plasticity can be defined across two scales at the plant level: within plant and among plant plasticity (Grossman & Rice, 2012), wherein plants of the same genotype exposed to different environments express different traits (Martin et al., 2018; Valverde‐Barrantes et al., 2013).

Often overlooked in these studies is the root system (Meyer & Purugganan, 2013). Yet, given the strong relationship between root trait expression and soil agroecosystem processes (e.g. carbon: De Deyn et al., 2008; nutrient cycling: Bardgett et al., 2014), how domestication has altered root trait expression is highly relevant to success in low input agriculture. It is hypothesized that modern crops may be unable to adequately shift root traits (i.e. express phenotypic plasticity) to maintain their yield rates when grown under different, and often more spatially and temporally heterogeneous, management regimes (Isaac et al., 2021; Rolhauser et al., 2022; Schmidt et al., 2016).

Shifts in root trait expression with domestication may be greater investment in the structural components of individual roots (e.g. greater root diameter, denser roots; Isaac et al., 2021) as opposed to prioritizing traits consistent with greater soil exploration and nutrient foraging (e.g. greater specific root length and surface area). Yet, it is unclear whether the shift in root functional trait expression with a crop history of domestication has come at the cost of root trait plasticity, as plasticity may be a heritable trait (Fitz Gerald et al., 2006; Sandhu et al., 2016), though is thought to be adaptive as well (Correa et al., 2019; Hodge, 2004). The retaining of root trait plasticity is a vital condition for crops to be able to capitalize on fertile pockets within heterogeneous soil environments (Borden et al., 2020; Grossman & Rice, 2012).

The current momentum towards replacing fertilizers with organic inputs presents crops with a very different set of soil conditions. While organic amendments may contain similar levels of nutrients to traditional synthetic fertilizers, they differ in the availability and the release rate (Iqbal et al., 2019; Rees & Castle, 2002), potentially leading to decreased yields for plants grown under organic management (Entz et al., 2018). Even over a short time, the use of organic amendments can alter the levels of soil organic carbon (C), nitrogen (N) and available phosphorous (P) (Herencia et al., 2008), while prolonged use is associated with changes to soil characteristics (e.g. soil aggregation and porosity; Domingo‐Olivé et al., 2016). The slow release rates of organic amendments over inorganic fertilizers provide substrates for microbial communities (Bastida et al., 2008) and shape the bioavailablity of nutrients and nutrient uptake efficiency for crops (Chen et al., 2022). Arguably, this altered nutrient availability and soil conditions linked with a potential increase in spatial heterogeneity of soil resources in organic systems will preferentially benefit a crop with a high degree of root trait plasticity, such that mean trait values shift based on localized conditions, as is beneficial for soil resource acquisition in less homogeneous environments.

An additional means of decreasing nutrient stress within organically managed environments is to intercrop with N_2_‐fixing legumes, drawing on atmospheric sources of N under limiting soil nutrient conditions, and contributing new N to soils via root exudation and, more significantly, via litter decomposition over subsequent growing seasons (Hauggaard‐Nielsen et al., 2008). Intercropping alters the available soil N by promoting the transfer of N from the roots of legumes to non‐legumes (Isaac et al., 2021; Jensen, 1996), which may also increase P availability due to soil acidification (Yan et al., 1996) and from root exudates of one intercropped species (Hinsinger et al., 2011). Similar to the use of organic amendments, intercropping may foster an increase in soil nutrient availability and microbial associations due to the multiple root systems present, which can benefit both species (Hinsinger et al., 2011). However, the benefits of intercropping can only be maximized if there is interspecific complementary (Yang et al., 2022), otherwise, it is presumed to not result in as many benefits given the potential competition for soil resources (Baumann et al., 2001).

In this study, spring wheat (*Triticum aestivum*) is our model crop species given its long history of recorded cultivation and widespread economic importance (Tuberosa et al., 2014). The wheat domestication syndrome is known to include increased growth rates (Matesanz & Milla, 2018), paired with greater total plant (Wacker et al., 2002) and aboveground biomass (Milla & Matesanz, 2017), though aboveground trait expression has shifted as a result of the Green Revolution (Austin et al., 1980). We employ a functional traits approach to determine if wheat with dissimilar domestication histories express different functional trait plasticity and how this expression changes under soil amendments and intercropping. We focus on both the degree of intraspecific variation exhibited by domesticated phenotypes and the direction of shifts in trait syndromes in response to altered soil environments. Our primary questions are: what is the extent of trait plasticity across varietals with different histories of domestication? How do soil amendments affect crop root trait expression? And is functional trait plasticity differentiated between species in intercropping systems? We expect that more recently developed wheat varietals will display constrained variation in root functional traits as a result of greater selective breeding for uniformity. We further expect that organic versus inorganic inputs will have differential effects on crop functional trait and agronomic trait expression, and while the strength of the response may be different, we expect the direction of the shifts in traits to be consistent regardless of domestication history. Additionally, these effects should be muted by intercropping with N_2_‐fixing crops as higher N availability will minimize soil nutrient availability effects.

## 2 METHODS

### 2.1 Study site

The field experiment was carried out at the University of Toronto Scarborough Campus Farm in Toronto, Ontario, Canada. Average air temperature, relative humidity and daily incoming PAR over the growing season were 19°C, 39% and 655 μmol m^−2^ s^−1^. The experimental area (~200 m^2^ of a multi‐acre site) was divided into 4.5 m × 4.5 m blocks, with each block containing a single amendment type. Two different soil amendment treatments were applied, an organic amendment (worm castings) and an inorganic fertilizer, both added at a rate of 10 t ha^−1^ (60 mg N per plant). Amendments were added 2 days prior to planting and mixed into the top 5 cm of soil. Each block was then divided into four 2 m × 2 m plots, where the intercropping treatment was applied. Each plot contained either monocropped wheat or wheat intercropped with soybean (*Glycine max*). Sub‐plots of 75 cm × 75 cm were demarked within a plot, each containing a different wheat varietal. The five varietals planted were Red Fife, Marquis, Neepawa, AAC Brandon and AAC Tradition (Table 1). Due to site constraints, most Brandon intercrops were planted in one block and the soil amendment treatment varied at the plot level. Wheat was planted in two 75 cm long rows within each sub‐plot, with 20 cm of space between rows, at a density of 35 seeds/row (124 plants m^−2^). Soybean planting followed 2 weeks after wheat planting. High nodulating soybeans were included and inoculated with Cell‐Tech Peat Soybean Inoculant prior to planting. Three 75 cm rows of soybeans were planted in intercropped sub‐plots, one in between and one on either side of the wheat rows with 20 cm between each soybean row.

**TABLE 1:** Histories of domestication for five wheat varietals.

Varietal	Year registered	Development goals	References
Red fife	1845	Ability to grow in Canadian soils, increased yields	Entz et al. (2018) and Symko (1999)
Marquis	1909	Decreased time to maturity, improved local adaptations, increased yield	Fu et al. (2005) and Morrison (1960)
Neepawa	1969	Increased fusarium head blight and stem rust resistance	Campbell (1970), Fu et al. (2005) and Zhu et al. (2019)
AAC Brandon	2013	Increased fusarium head blight and leaf rust resistance, increased yields, larger seed size	Entz et al. (2018) and Zhu et al. (2019)
AAC Tradition	2016	Developed under organic management for yield, height, maturity and disease resistance	Entz et al. (2018) and Government of Canada (n.d.)

Soil samples were taken for nutrient analysis. Soil for N and C analysis was oven‐dried at 105°C for 48 h, ground with a ball mill (Retsch Ltd., Haan, Germany) and analyzed in the LECO elemental analyzer (LECO Instruments, Mississauga, ON, Canada). Plots amended with inorganic fertilizer (C = 6.57% ± 1.47, N = 0.35% ± 0.11, C:N = 19.21 ± 3.06) had similar mean soil C and N values as those amended with organic amendment (C = 8.76% ± 1.88, N = 0.52% ± 0.12, C:N = 17.01% ± 1.21) at the beginning of the experiment.

### 2.2 Plant sampling and analysis

Sampling was conducted when 50% of each wheat variety had fully ripened spikes, at the beginning of October 2020. Within each sub‐plot (*n* = 60), all wheat and soybean plant roots were fully excavated so the whole root systems were extracted. The number of wheat and soybean plants was counted and recorded. Two representative wheat plants and three soybean plants were separated for further sampling. From each of the two samples of wheat, a young fully emerged leaf was cut from the stem, thickness was measured (mm) and the leaf was photographed to assess leaf area (LA, cm^2^) using ImageJ prior to being dried for 48 h at 65°C and weighed to obtain leaf dry mass, used to calculate specific leaf area (SLA, cm^2^ g^−1^). An intact lateral root was removed from these two wheat samples and one soybean plant from each sub‐plot and placed in separate plastic bags. Samples were stored in a refrigerator before being rinsed in deionized water and scanned using a flatbed scanner at 600 dpi. Root scans were analyzed for morphological metrics using WinRhizo (Regents Instruments, Montreal, QC, Canada). The lateral roots were then dried for 48 h at 65°C, and weighed for dry mass and used in conjunction with the root length to calculate specific root length (SRL, m g^−1^) and specific root area (SRA, m^2^ g^−1^). The two wheat plants from each sub‐plot were then separated into three agronomic trait components [roots, aboveground biomass (stems and leaves) and spikes]. For soybeans, beans, shoots, roots and nodules were removed and dried for 48 h at 65°C prior to weighing. Leaf and root C and N concentrations (mass %) were determined with a LECO elemental analyzer after being dried and ground with a ball mill. All remaining shoot, root and spike components were dried for 48 h at 65°C and weighed. The remaining wheat plants from each sub‐plot were grouped together and then separated into roots, aboveground biomass, and spikes and dried and weighed.

### 2.3 Statistical analyses

All statistical analyses were performed in R v. 4.2.0 (R Foundation for Statistical Computing, Vienna, Austria). Significance levels were set at *p* < .05. Descriptive statistics were calculated for trait and yield data for wheat and soybeans across intercropping treatments and soil amendment treatments and were tested for normality using a maximum likelihood approach, comparing models based on log‐likelihood ratios. Traits were log‐transformed if best described by log‐normal distributions. Initial analysis on the differences in mean functional trait expression due to wheat varietal, amendment and intercropping treatment effects were analyzed using three‐way ANOVAs, with plots as a random effect to account for pre‐existing soil variability potentially present at the site. We coupled these models with a Tukey post hoc test using the ‘emmeans’ package assess differences in functional traits across the soil amendment*intercropping*varietal design.

We performed a permutation analysis of variance (PerMANOVA) on wheat leaf and root functional traits based on 999 permutations, to test differences in multivariate trait relationships between wheat varietals, soil amendment and intercropping treatments. Functional trait hypervolumes were constructed using the ‘hypervolume’ R package and a Gaussian kernel density estimate (Blonder et al., 2018). We created five‐dimensional hypervolumes for each wheat varietal planted in monocrop, combining both soil amendment treatments. Before analyses, all measured data was standardized by z‐transformation (i.e. to zero mean and unit variance) to compare axes with different units. Leaf trait hypervolumes included the five functional traits (LA, SLA, leaf thickness, leaf N, leaf CN) and root trait hypervolumes included the five root functional traits collected (SRL, SRA, average root diameter, root N, root CN). The data collected during the experiment only allowed for the creation of one robust hypervolume each for leaf and root systems, and therefore, repeated simulations of trait data were created. Using the ‘replicate’ R function, five replicate datasets were created using the mean and standard deviation for each varietal's traits so that statistical tests could be performed. Volumes were built from the modelled data. The visual analysis of the hypervolumes was based on the observed, not modelled, data. We tested for differences in total volume using a one‐way ANOVA. To determine whether niche partitioning occurred in trait space between wheat and intercropped soybean, we further created root trait hypervolumes which included all intercropped wheat and soybean using the same root traits as previously. We similarly tested differences between species for volume using the same replicate function. We reported Jaccard similarity and centroid distances between the species and unique hypervolume percentages.

## 3 RESULTS

### 3.1 Crop trait variation

Across the entire data set, leaf and root morphological traits had higher variability than leaf and root chemical traits, with coefficients of variation ranging from 18.55 (leaf thickness) to 67.10 (root weight), while leaf N and root N were cv < 20 (Table S1). Yet, the most highly variable wheat traits were yield metrics (spike weight per plant: cv = 69.85, average single spike weight: cv = 39.32) and shoot (cv = 50.81) and root weights (cv = 67.10), whereas soybean variability was greatest for functional traits SRL (cv = 67.26) and SRA (cv = 76.38), followed by nodule (cv = 69.30) and bean weight (cv = 53.87).

### 3.2 Treatment effects on crop functional traits

**FIGURE 1:**
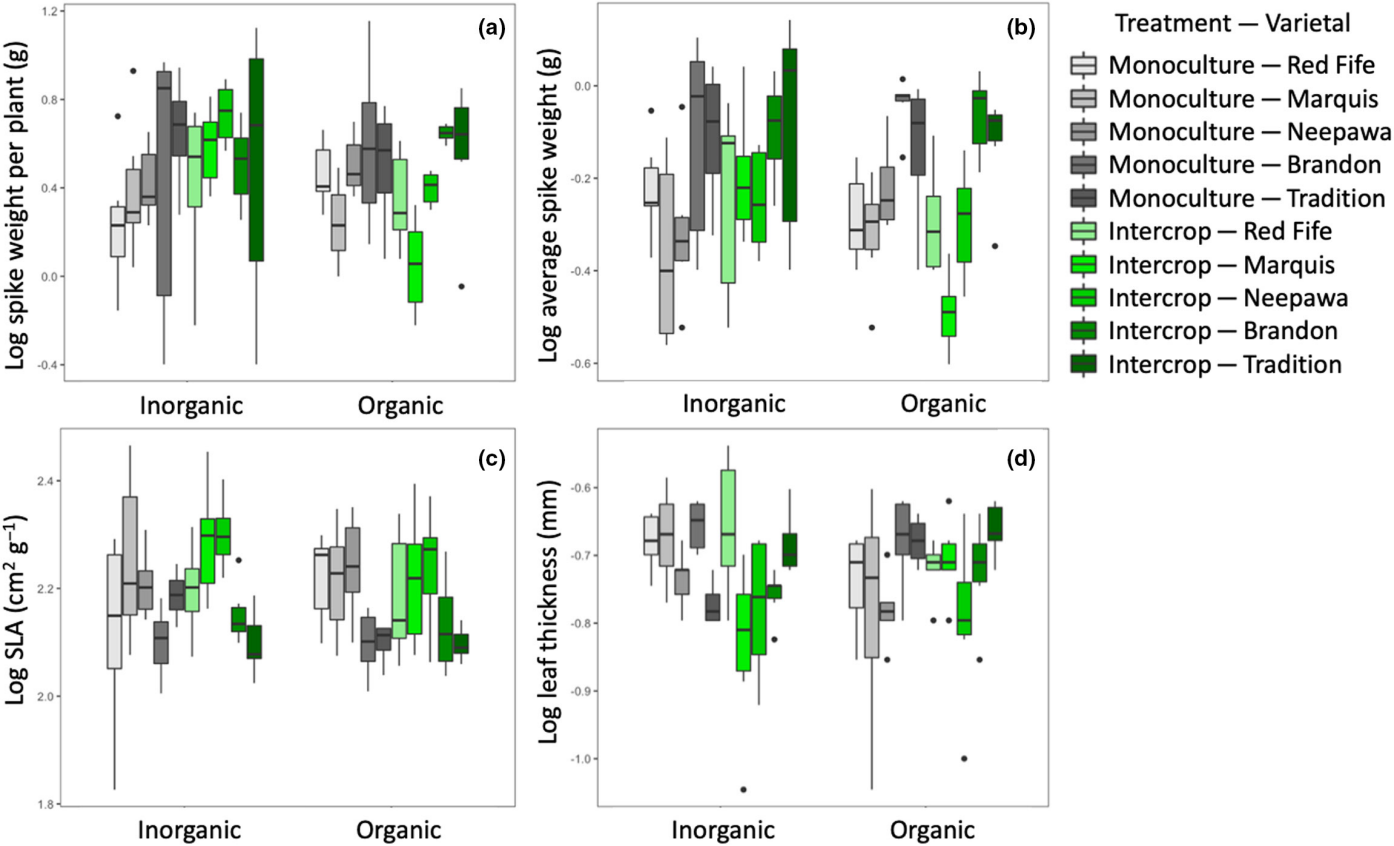
Yield and leaf trait values (a: Log spike weight per plant, b: Log average spike weight, c: Log SLA and d: Log leaf thickness) for five wheat varietals planted with inorganic and organic amendments, under intercropping or monocropping planting designs.

**FIGURE 2:**
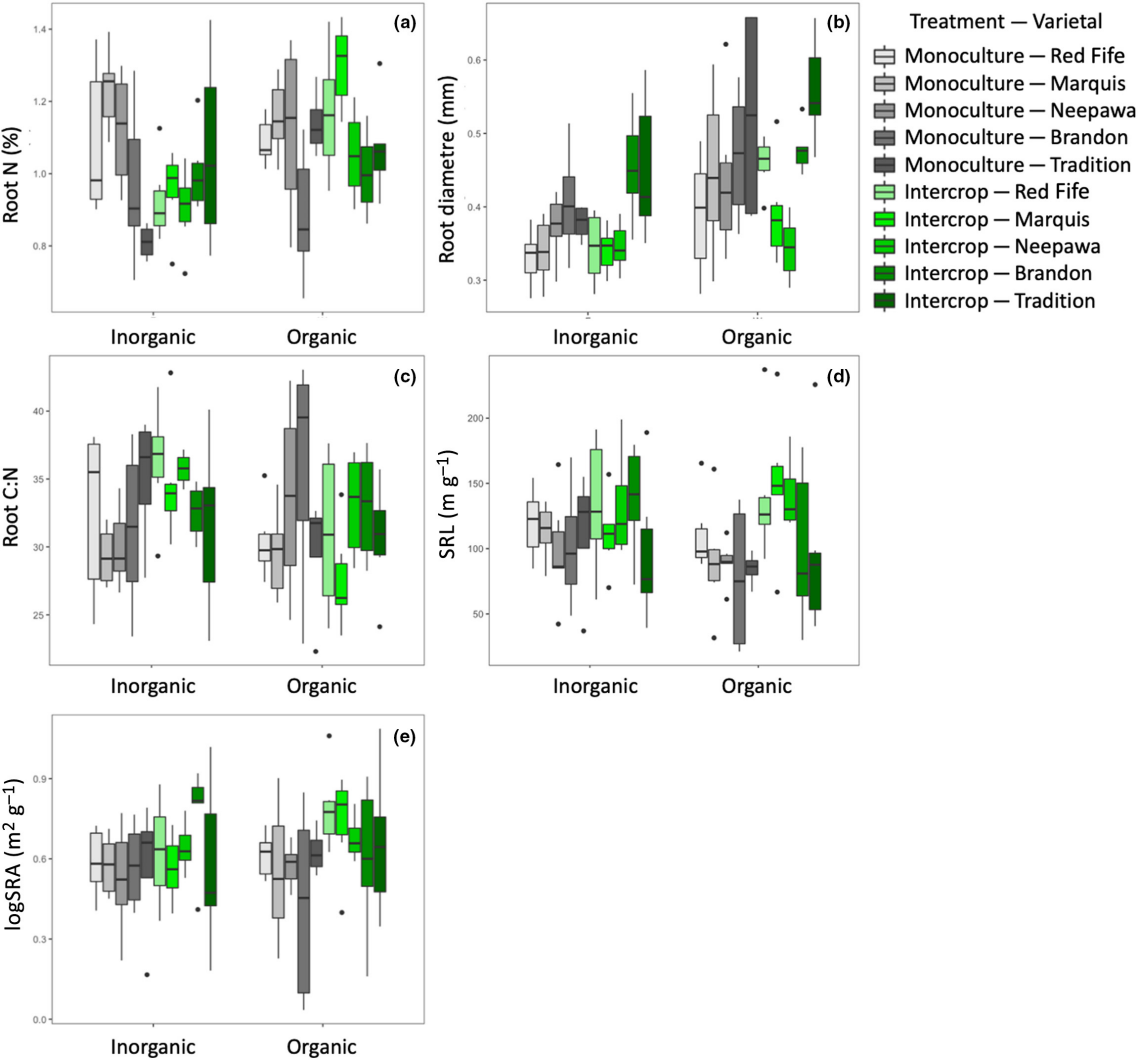
Root trait values (a: root N, b: root diameter, c: root CN, d: SRL, e: Log SRA) for five wheat varietals planted with inorganic and organic amendments, under intercropping or monocropping planting designs.

Two‐way ANOVA results for the five leaf functional traits showed that SLA (Figure 1c, *p* < .001) and leaf thickness (Figure 1d, *p* = .002) varied with varietal, while leaf N and CN did not (Table 2). Intercropping with soybean led to significantly greater values of wheat SLA (*p* = .007 Figure 1c). Yield traits (spike weight per plant: *p* = .001, average single spike weight: *p* < .001, Table 2, Figure 1a,b) varied significantly with varietal, while the soil amendment*varietal interaction was significant for spike weight per plant (*p* = .023). Shoot weight was not significantly variable with any of the model terms. Of the five root traits, all but SRL and SRA varied significantly with varietal (average diameter: *p* < .001; root N: *p* < .001; root CN: *p* = .042, Figure 2a–c, Table 3). SRL (*p* = .002) and SRA (*p* = .004) were both significantly greater under intercropping (Figure 2d,e, Table 3). The soil amendment treatment had no effect on root trait expression.

**TABLE 2:** ANOVA results for agronomic and leaf trait data of five wheat varietals with intercropping (intercropped with soybean and monocrop wheat) and amendment treatments (inorganic fertilizer, organic amendment).

	df	Agronomic traits			Leaf traits				
		Spike weight per plant	Single spike weight	Shoot weight	LA	SLA	Leaf thickness	Leaf N	Leaf CN
Amendment	1	0.498 (0.482)	0.014 (0.906)	1.150 (0.286)	1.325 (0.253)	0.094 (0.760)	0.226 (0.635)	1.974 (0.164)	1.229 (0.271)
Intercropping	1	0.371 (0.544)	3.342 (0.071)	0.489 (0.486)	1.411 (0.238)	**7.651 (0.007)**	1.170 (0.282)	0.243 (0.623)	0.302 (0.584)
Varietal	4	**5.504 (0.001)**	**17.792 (<0.001)**	1.031 (0.396)	1.881 (0.121)	**12.271 (<0.001)**	**4.629 (0.002)**	1.022 (0.401)	1.423 (0.233)
Amendment:Intercropping	1	0.689 (0.409)	1.744 (0.190)	0.068 (0.794)	0.048 (0.828)	0.263 (0.609)	2.117 (0.149)	0.067 (0.796)	0.005 (0.945)
Amendment:Varietal	4	**3.00 (0.023)**	1.166 (0.331)	2.234 (0.072)	1.444 (0.226)	1.612 (0.178)	1.775 (0.141)	1.183 (0.324)	1.439 (0.228)
Intercropping:Varietal	4	0.506 (0.731)	0.559 (0.693)	0.582 (0.676)	1.023 (0.400)	0.481 (0.750)	1.327 (0.266)	1.767 (0.143)	1.576 (0.188)
Amendment:Intercropping:Varietal	4	0.404 (0.805)	1.619 (0.176)	1.089 (0.367)	2.099 (0.087)	0.632 (0.641)	**3.436 (0.012)**	1.036 (0.393)	0.855 (0.495)

*Note*: *F* values are reported and *p* values are in brackets, bold denotes significance.

**TABLE 3:** ANOVA results for root trait data of five wheat varietals with intercropping (intercropped with soybean and monocrop wheat) and amendment treatments (inorganic fertilizer, organic amendment).

	df	Root weight	Average diameter	SRL	SRA	Root N	Root CN
Amendment	1	0.018 (0.892)	0.309 (0.430)	0.349 (0.556)	0.000 (0.984)	0.021 (0.886)	0.698 (0.406)
Intercropping	1	0.001 (0.976)	0.315 (0.576)	**10.010 (0.002)**	**8.640 (0.004)**	0.025 (0.874)	0.886 (0.349)
Varietal	4	**5.515 (0.001)**	**12.017 (<0.001)**	1.904 (0.117)	0.814 (0.520)	**11.062 (<0.001)**	**2.599 (0.042)**
Amendment:Intercropping	1	1.622 (0.206)	2.878 (0.093)	1.792 (0.184)	0.690 (0.409)	2.084 (0.152)	0.946 (0.333)
Amendment:Varietal	4	1.143 (0.342)	1.477 (0.216)	0.558 (0.694)	1.284 (0.282)	0.541 (0.706)	1.352 (0.257)
Intercropping:Varietal	4	0.345 (0.847)	**2.966 (0.024)**	0.835 (0.506)	0.373 (0.827)	0.880 (0.479)	0.602 (0.662)
Amendment:Intercropping:Varietal	4	0.824 (0.513)	0.908 (0.463)	0.439 (0.780)	0.279 (0.891)	**3.310 (0.014)**	0.964 (0.432)

*Note*: *F* values are reported and *p* values are in brackets, bold denotes significance.

We used PerMANOVAs to assess the effects of varietal, soil amendment and intercropping treatments on multivariate trait space. We found that for leaf traits, plot (*R* ^2^ = .04, *p* = .02) and soil amendment (*R* ^2^ = .04, *p* = .009) were significant model terms, while varietal (*R* ^2^ = .06, *p* = .08) and intercropping were not (*R* ^2^ = −.01, *p* = .98). A subsequent pairwise test found that Red Fife had significantly different multivariate trait relationships than AAC Tradition (*F* = 5.60, *p* = .01). Root multivariate traits were significantly affected by plot (*R* ^2^ = .04, *p* = .02) and intercropping (*R* ^2^ = .05, *p* = .01), while varietal (*R* ^2^ = .06, *p* = .06) and soil amendment were not (*R* ^2^ = .01, *p* = .53). A subsequent pairwise test found differences between both newer varietals and Red Fife (Brandon: *F* = 3.66, *p* = .04; Tradition: *F* = 5.98, *p* = .01).

### 3.3 Trait hypervolume variation

**FIGURE 3:**
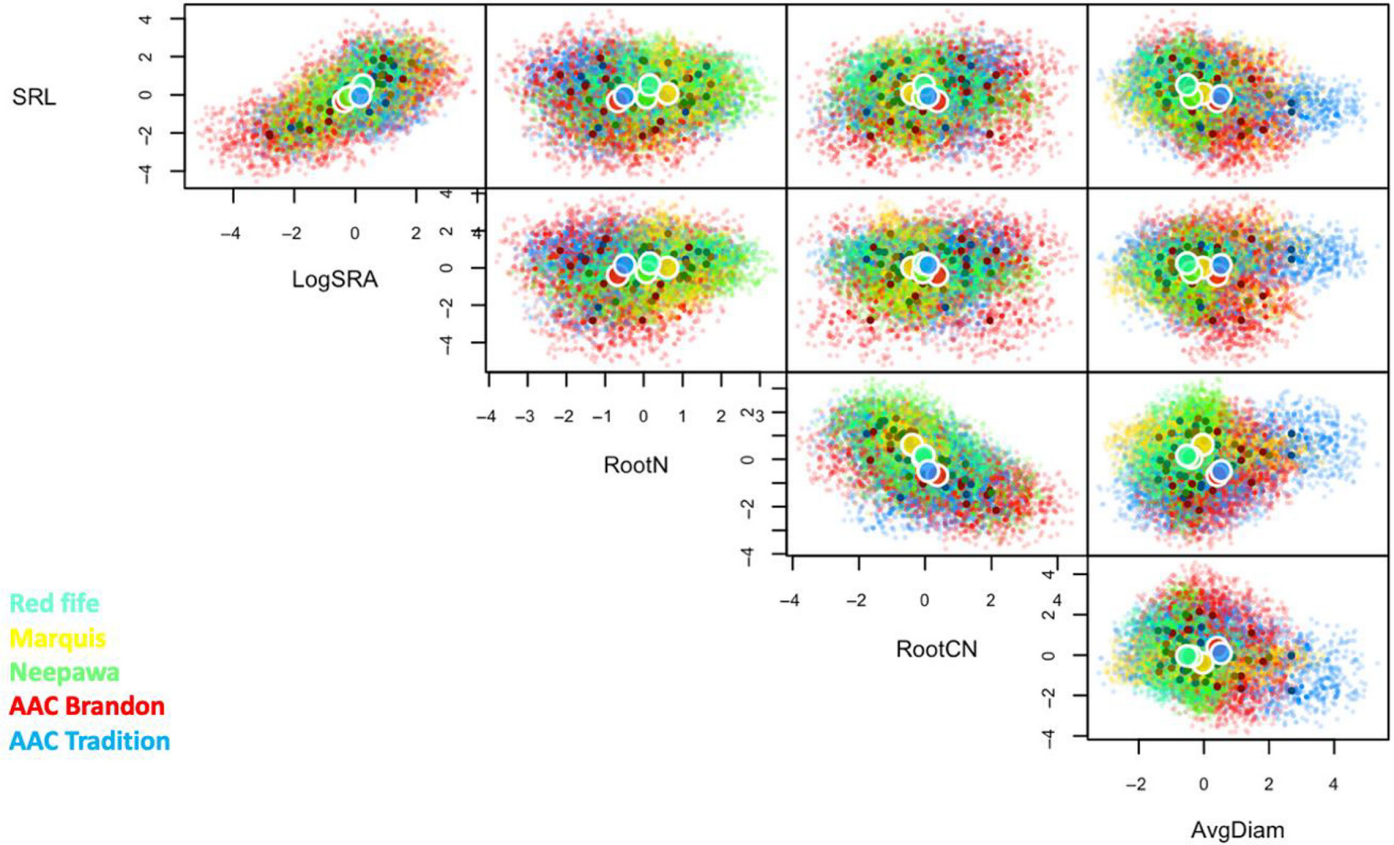
Root trait hypervolumes for multiple bivariate functional trait axes (SRL: specific root length, LogSRA: log‐specific root area, RootN: root N %, RootCN: root C:N, AvgDiam: average root diameter) in five wheat varietals planted alone across different amendments (inorganic fertilizer and organic amendment).

**FIGURE 4:**
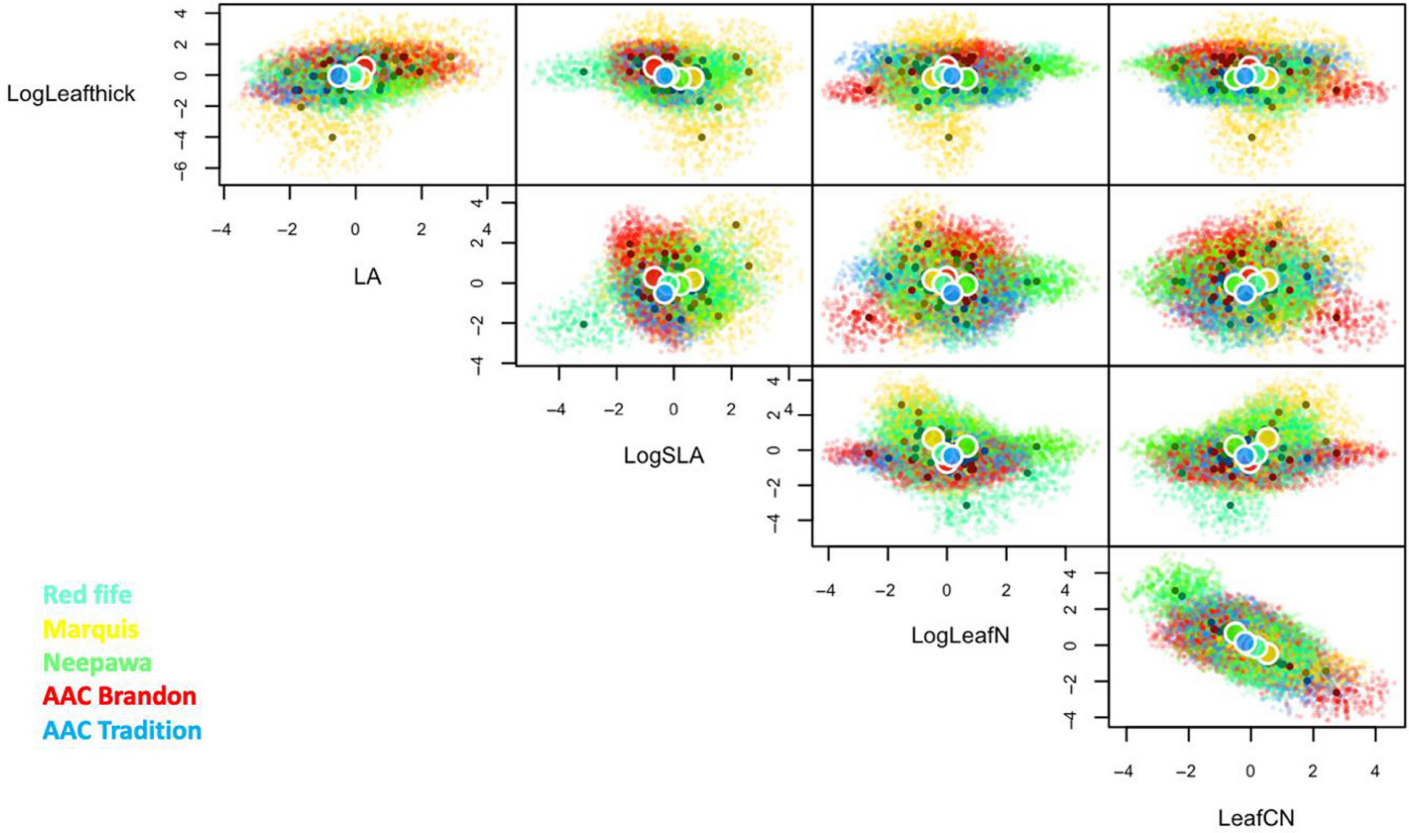
Leaf trait hypervolumes for multiple bivariate functional trait axes in five wheat varietals planted alone in two different amendments (inorganic fertilizer and organic amendment).

**FIGURE 5:**
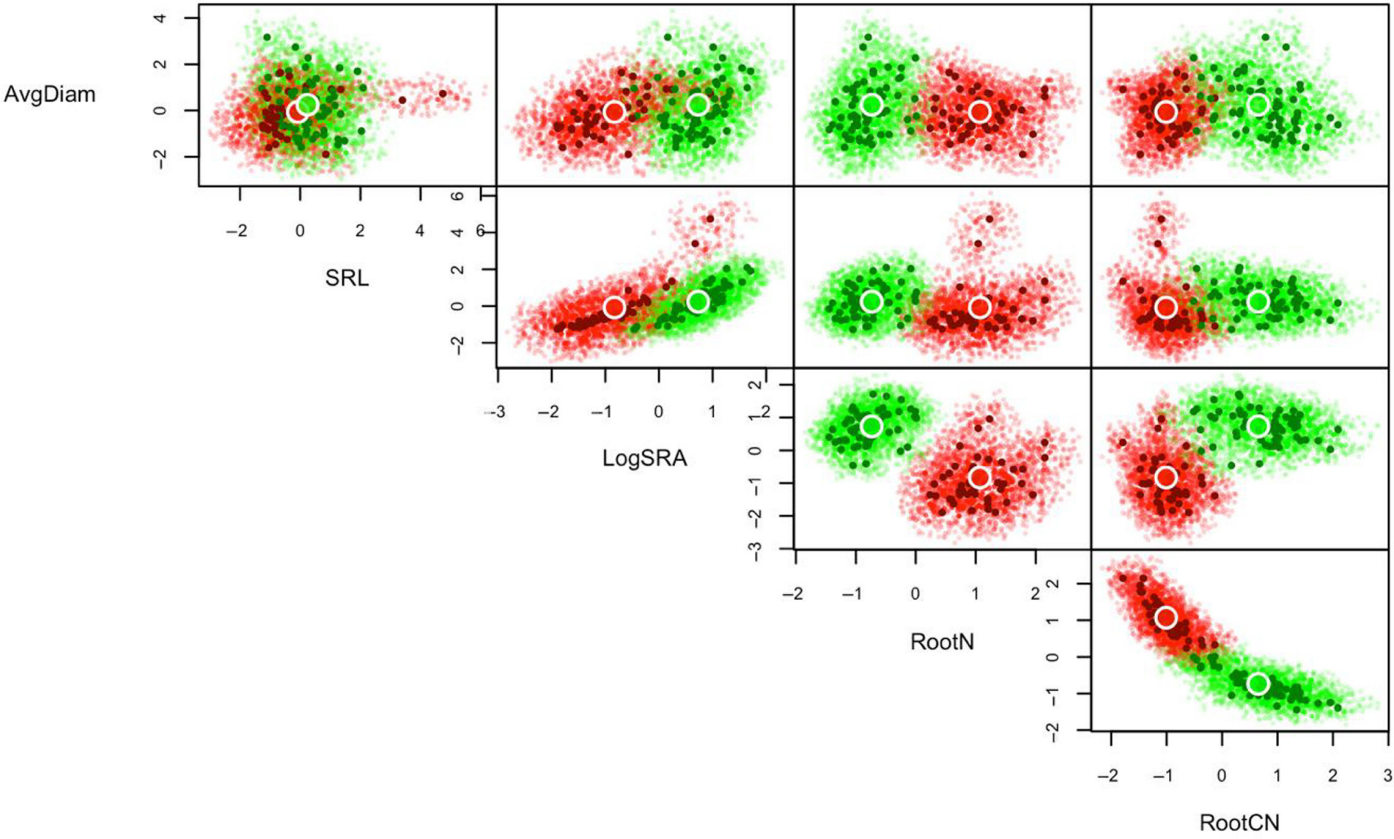
Root trait hypervolumes for multiple bivariate functional trait axes (SRL: specific root length, LogSRA: log specific root area, RootN: root N %, RootCN: root C:N, AvgDiam: average root diameter) for wheat (green) and soybean (red) intercropped with different amendment treatments (inorganic fertilizer and organic amendment).

We used five‐dimensional trait hypervolume analysis to visualize crop trait space for wheat roots (Figure 3) and leaves (Figure 4) in monoculture as well as for intercropped wheat and soybean (Figure 5), using the additional modelled data for statistical purposes (Table 4). Modelled trait hypervolumes for wheat in monoculture showed significantly different volumes for root traits between wheat varietals (*F* = 9.48, *p* < .001, Figure 3). Specifically, Red Fife had a smaller hypervolume than AAC Tradition (*p* = .05), while AAC Brandon had a significantly larger hypervolume than Marquis (*p* < .001), Neepawa (*p* < .01) and Red Fife (*p* < .001). Leaf trait hypervolume sizes in wheat were significantly different between varietals (Figure 4, *F* = 4.89, *p* < .001). Specifically, Marquis had a larger hypervolume than AAC Brandon (*p* = .05), Neepawa (*p* = .01) and AAC Tradition (*p* = .01). When all varietals of intercropped wheat were analyzed with soybean, we found that the trait volume did not differ significantly between the species (*F* = 5.32, *p* = .08, Figure 5, Table 4). However, we found that the hypervolume space occupied within the 5D volume was very different between the two species. We found average Jaccard and Sorensen scores of <0.001 ± <0.001, both denoting very little overlap between the trait hypervolumes for wheat and soybean when intercropped. The average unique hypervolume fractions for both species were almost the entirety of the hypervolume space (wheat: 99.9% ± 0.1; soybean: 99.9% ± 0.01).

**TABLE 4:** Hypervolume size (±SD) for five roots (SRL, SRA, root N, root CN, root weight) and five leaf traits (LA, SLA, leaf thickness, leaf N, leaf CN) for monocropped wheat varietal hypervolumes, and root trait hypervolume size (±SD) for intercropped soybean and wheat.

	Root hypervolume	Leaf hypervolume
Varietal		
Red Fife	344.64 (183.82)	371.47 (224.19)
Marquis	460.97 (243.50)	621.04 (377.26)
Neepawa	694.08 (372.29)	125.66 (75.95)
AACBrandon	3929.87 (2130.52)	222.13 (134.34)
AAC Tradition	2488.01 (1328.65)	127.75 (76.85)
Species		
Soybean	112.00 (69.11)	
Wheat	44.68 (33.08)	

## 4 DISCUSSION

### 4.1 Trait plasticity in crops with different histories of domestication

Direct selection pressures which formed the domestication syndrome of wheat and other grasses have focused on aboveground trait expression, suggesting trait syndromes of larger leaves and greater growth rates (Gómez‐Fernández et al., 2022; Milla et al., 2014; Milla & Matesanz, 2017), while little direct pressure was applied to root systems (Meyer et al., 2012). Previous studies on wheat root trait expression typically rely on fewer traits (eg. Duncan et al., 2018), exclusively modern varietals (eg. Djanaguiraman et al., 2019), or growth chamber grown specimens for extensive root analysis (eg. Friedli et al., 2019; Junaidi et al., 2018). In our field study with a suite of varieties, the overall wheat trait variation was quite large, especially for morphological traits, complementing the results of others for wheat (leaf: Martin et al., 2018, and root: Friedli et al., 2019; Iannucci et al., 2021). However, we did find greater differences in chemical traits than Cantarel et al. (2021), who report a root N % cv of ~0.4 compared to our cv of 17 across wheat varietals.

For agronomic and leaf traits, modern varietals, AAC Brandon and AAC Tradition, expressed greater yield and specific leaf area values regardless of soil amendment treatment or whether they were under intercropping, results that are in line with their expected domestication syndromes. Breeding for higher yield and fitness in optimum conditions may be associated with higher performance in more stressful conditions (Mercer & Perales, 2010; Sadras & Denison, 2016), and our findings support this pattern. However, wheat roots generally expressed more conservative traits with domestication; modern varieties had greater average root diameter and higher root CN values. These opposing shifts along an economic spectrum in leaf and root traits with domestication were also recently found in a data review (Isaac et al., 2021). This decoupling of resource acquisition patterns in crop leaves and roots is not uncommon (Isaac et al., 2017) and could be a result of relaxed trait relationships in crops (Martin & Isaac, 2015), especially with domestication (Milla et al., 2014; Roucou et al., 2018).

While currently debated (see: Gaudin et al., 2011; Grossman & Rice, 2012; Jaradat, 2018; Matesanz & Milla, 2018), it has been proposed that domestication could lead to decreased phenotypic plasticity as a result of breeding for uniformity. Using an analysis of trait space as a proxy of trait plasticity with domestication history, we found contrasting evidence in regard to this hypothesis. At the leaf level, more recently domesticated varieties AAC Brandon and AAC Tradition, both developed only in the past decade (Table 1), occupied smaller leaf trait spaces (Figure 4), but at the root level, they occupied larger root trait spaces (Figure 3), which suggests that root trait plasticity is retained and indeed potentially larger for modern wheat varietals (contrary to expectations, eg. Matesanz & Milla, 2018). These findings suggest that breeding for aboveground uniformity (which is confirmed by our leaf trait plasticity results) has not led to a concurrent decrease in the ability of a plant to vary trait expression in roots in response to altered environmental pressures. This increased plasticity with domestication could be due to greater genomic plasticity resultant from increased hybridization or gene introduction events in more modern varietals (Rajpal et al., 2016). But overall the findings largely suggest that while wheat roots tend to shift toward conservative traits in modern varieties, their potential to adapt to environmental conditions is greater than wheat leaves.

### 4.2 Trait complementarity between intercropped species

We show that wheat root trait space has minimal overlap with soybean root trait space when intercropped and grown under various soil amendment regimes, supporting previous findings on trait coordination (Ajal et al., 2021). Interestingly, there was little difference between conservative root trait expression of wheat grown in monoculture and wheat grown in intercropping; root diameter, root N and root CN were not significantly different under intercropping and monocropping treatments. This suggests that wheat in our experiment exhibits natural trait complementarity, where species occupy different trait niches naturally, and exhibit little actual shift in trait expression in order to minimize competition in resource use (Hauggaard‐Nielsen et al., 2008; Isaac et al., 2021). However, these traits were strongly influenced by domestication history, thus providing evidence that conservative traits are more controlled by domestication rather than the local environment.

The intercropping treatment was however found to be a significant source of variation in wheat for leaf (SLA) and root (SRA, SRL) acquisitive traits. In fact, wheat intercropped with soybean consistently expressed greater resource acquisition strategies (higher SLA, SRA, and SRL) than wheat in monoculture, regardless of variety. Greater root length and root surface area in wheat intercropped with soybean was previously found, and the authors also highlight the importance of root depth when assessing root trait complementarity in intercropped systems (Bargaz et al., 2017). Furthermore, higher soil volume exploration under intercropping with soybean was also detected for maize under N limited conditions (Yang et al., 2022). Presumably, greater soil exploration, via expression of acquisitive root traits, is a common signature of intercropped non‐leguminous species. In our study, we found that these root functional traits only varied with intercropping and did not change with domestication history nor with type of soil amendment.

For agronomic traits, we found that both the average spike weight and total spike weight per plant were unchanged across intercropping treatments. Given the additive design of our intercropping study, the unchanged yield per plant in intercropped treatments vs monocrops suggests that there is a net benefit (ie. a land equivalent ratio of over 1, Willey & Osiru, 1972) for these wheat varietals to intercropping. This is somewhat contrasted by the results of Ajal et al. (2021), who used inorganic fertilizers and wheat‐faba bean intercrops and found wheat yield was in fact greater in intercropped plots, or by the results of Pridham and Entz (2008) who found that under organic management wheat yields decreased when intercropped with a range of leguminous species.

### 4.3 Root functional trait expression in response to organic amendments

While we hypothesized that soil amendments would be a primary driver of differences in wheat root trait expression, we found a relatively small degree of difference in root trait expression in response to soil amendment type. Specifically, we expected to see differences in trait expression of chemical traits, as roots may uptake the elevated bioavailable N from inorganic fertilizer, and translocate to leaves. The lack of chemical trait response to the amendment treatments could be due in part to the very small differences in soil N at the end of the growing season (organic amendment: soil total N = 0.52% ± 0.12, inorganic fertilizer: soil total N = 0.35% ± 0.11), thus minimizing differences between the two amendments. Additionally, there is evidence of differential rates of N translocation to grain yields between wheat varieties (Arduini et al., 2006; Osman et al., 2012), which may be masking any N content differences present due exclusively to soil amendment type.

Similarly, while soybean root trait expression was not significantly different between soil amendment types, we did detect higher average nodule mass in soybeans amended with inorganic fertilizer (Table S2). This suggests that wheat may be competitively acquiring soil N under inorganic fertilization (Gagnon et al., 1997), thus reducing available N sources for neighbouring soybean and stimulating soybean to shift to energy sources to nodule formation and N_2_ fixation in this limited N environment (Schipanski et al., 2010), earlier in the growing season. In organic conditions, both wheat and soybean may have similar competitive advantages given the organic forms of N and the need for mineralization, however, wheat may be more competitive than soybean at taking up inorganic N sources (Li et al., 2001).

## 5 CONCLUSIONS

In this study, using a suite of wheat varietals with different histories of domestication, we tested for variation in leaf and root functional traits (i) in relation to domestication history and (ii) as moderated by organic amendments and intercropping with soybean. We found that the year of wheat varietal release, a proxy for their history of domestication explains functional trait variation, and, importantly, that intercropping rather than soil amendments result in larger shifts in wheat root functional trait expression. In fact, while wheat roots expressed conservative traits and a larger trait space with domestication, all wheat varieties tended to express more acquisitive root traits when intercropped. The strong relationship between intraspecific trait variation and variety underscores the need to consider phenotypic suitability in different growing systems in order to enhance growth potential. Yield variation resulting from intercropping with soybean suggests that some varietals may be better suited and able to express beneficial trait trade‐offs in competitive environments. Understanding root trait variability from a functional ecology and evolutionary perspective will contribute to breeding programs to develop seeds optimized for transitions to diversified and organic agriculture.

## AUTHOR CONTRIBUTIONS

**Victoria Nimmo:** Conceptualization (lead); data curation (lead); formal analysis (lead); investigation (lead); methodology (lead); writing – original draft (lead). **Cyrille Violle:** Formal analysis (equal); writing – review and editing (equal). **Martin Entz:** Writing – review and editing (equal). **Andres G. Rolhauser:** Formal analysis (equal); visualization (equal); writing – review and editing (equal). **Marney E. Isaac:** Conceptualization (equal); formal analysis (equal); funding acquisition (lead); methodology (equal); project administration (equal); supervision (lead); writing – review and editing (equal).

## FUNDING INFORMATION

We acknowledge the Natural Sciences and Engineering Research Council of Canada, the Canada Research Chairs program and the Sustainable Food and Farming Futures Cluster for financial support.

### OPEN RESEARCH BADGES

This article has earned Open Data, Open Materials and Preregistered Research Design badges. Data, materials and the preregistered design and analysis plan are available at [https://doi.org/10.5061/dryad.h9w0vt4nb].

## Supporting information


Table S1.
Table S2.

## Data Availability

Raw data that support the findings of this study are openly available in DRYAD at https://doi.org/10.5061/dryad.h9w0vt4nb.
