# Supplementary material for: Changes in crop trait plasticity with domestication history: Management practices matter
Source: Ecol Evol. 2023 Nov 15;13(11):e10690. doi: 10.1002/ece3.10690 (PMC10651313; doi:10.1002/ece3.10690)
Supplement: Supplementary file 1 — Table S1. Table S2. [file ECE3-13-e10690-s001.docx]

**Supplementary Data**

**Table S1.** Variation in yield, leaf, stem, and root functional traits for wheat, and for yield, stem, and root traits for soybean. Maximum likelihood best fits are bolded.

|  | Normal maximum likelihood | Log maximum likelihood | Mean | SD | Range | CV |
| --- | --- | --- | --- | --- | --- | --- |
| Wheat traits | | | | | | |
| Leaf and yield | | | | | | |
| Spike weight/plant (g) | -272.68 | **-251.19** | 3.65 | 2.55 | 0.40-14.30 | 69.85 |
| Average single spike weight (g) | -7.25 | **-1.80** | 0.66 | 0.26 | 0.25-1.39 | 39.32 |
| Leaf thickness (mm) | 222.48 | **214.42** | 0.19 | 0.03 | 0.09-0.29 | 18.55 |
| Leaf area (cm^2^) | **-320.36** | -326.39 | 16.99 | 3.85 | 5.88-29.98 | 22.63 |
| SLA (cm^2^g^-1^) | -595.00 | **-587.00** | 157.57 | 41.05 | 67.06-292.06 | 26.05 |
| Shoot weight (g) | -308.49 | **-301.41** | 6.83 | 3.47 | 0.70-20.70 | 50.81 |
| LeafN (%) | -58.40 | **-53.53** | 3.21 | 0.40 | 2.25-4.79 | 12.62 |
| Leaf CN | **-215.28** | -216.31 | 13.87 | 1.61 | 9.48-18.77 | 11.58 |
| Root | | | | | | |
| Root weight (g) | -148.00 | **-112.73** | 1.30 | 0.87 | 0.29-5.64 | 67.10 |
| Average root diameter (mm) | **114.31** | 122.08 | 0.41 | 0.09 | 0.27-0.66 | 22.03 |
| SRL (mg^-1^) | **-605.42** | -613.28 | 112.29 | 44.90 | 21.22-237.20 | 39.99 |
| SRA (m^2^g^-1^) | -245.28 | **-237.85** | 4.54 | 2.01 | 1.08-12.21 | 44.38 |
| RootN (%) | **33.59** | 33.73 | 1.05 | 0.18 | 0.65-1.43 | 17.18 |
| RootCN | **-342.93** | -343.57 | 32.17 | 4.92 | 22.31-43.05 | 15.30 |
| Soybean traits | | | | | | |
| Leaf and yield | | | | | | |
| Shoot weight (g) | **16.70** | 19.52 | 0.42 | 0.14 | 0.20-0.83 | 33.67 |
| Bean weight/plant (g) | 18.35 | **11.84** | 0.25 | 0.13 | 0.02-0.61 | 53.87 |
| Root | | | | | | |
| RootN (%) | **2.31** | 2.94 | 1.90 | 0.23 | 1.54-2.38 | 11.98 |
| RootCN | -70.71 | **-70.04** | 19.67 | 2.60 | 15.76-25.98 | 13.21 |
| Average root weight (g) | 45.02 | **43.42** | 0.20 | 0.05 | 0.08-0.36 | 27.26 |
| Average nodule weight (g) | **91.23** | 95.26 | 0.02 | 0.01 | 0.003-0.047 | 69.30 |
| Average root diameter (mm) | **39.83** | 39.97 | 0.39 | 0.06 | 0.26-0.54 | 16.58 |
| SRL (mg^-1^) | -231.49 | **-217.83** | 82.12 | 55.24 | 31.5-317.54 | 67.26 |
| SRA (m^2^g^-1^) | -104.12 | **-88.87** | 10.362 | 7.91 | 3.52-44.11 | 76.38 |

**Table S2.** ANOVA results for soybean yield and trait data when intercropped with 5 different wheat varietals and planted in two amendment treatments (inorganic fertilizer, organic amendment). F-values are reported and p values are in brackets, bolding denotes a significant response.

|  | Amendment | Wheat varietal | Amendment:Wheat varietal |
| --- | --- | --- | --- |
| Df | 1 | 4 | 4 |
| Average shoot weight | 0.0001 (0.982) | 0.942 (0.468) | 0.872 (0.505) |
| Log average bean weight | 1.050 (0.323) | 1.115 (0.388) | 0.044 (0.996) |
| Root N | 0.507 (0.488) | 1.892 (0.168) | 0.657 (0.632) |
| Log root CN | 0.050 (0.825) | 1.234 (0.341) | 0.603 (0.667) |
| Log average root weight | 0.019 (0.893) | 0.869 (0.507) | 0.660 (0.630) |
| Average nodule weight | **7.692 (0.015)** | 1.029 (0.426) | 0.944 (0.467) |
| Average diameter | 1.482 (0.243) | 1.089 (0.399) | 0.775 (0.559) |
| Log SRL | 0.196 (0.665) | 0.436 (0.780) | 0.422 (0.790) |
| Log SRA | 0.098 (0.758) | 0.700 (0.604) | 0.672 (0.622) |
